# Sirt6 promotes tumorigenesis and drug resistance of diffuse large B-cell lymphoma by mediating PI3K/Akt signaling

**DOI:** 10.1186/s13046-020-01623-w

**Published:** 2020-07-25

**Authors:** Juan Yang, Ying Li, Ya Zhang, Xiaosheng Fang, Na Chen, Xiangxiang Zhou, Xin Wang

**Affiliations:** 1 Department of Hematology, Shandong Provincial Hospital Affiliated to Shandong University, Shandong First Medical University, No.324 Jingwu Road, Jinan, 250021 Shandong China; 2 School of Medicine, Shandong University, Jinan, 250012 Shandong China; 3 Shandong Provincial Engineering Research Center of Lymphoma, Jinan, 250021 Shandong China; 4 National clinical research center for hematologic diseases, Jinan, 250021 Shandong China

**Keywords:** Diffuse large B-cell lymphoma, Sirt6, OSS_128167, Oncogene, Therapeutic target

## Abstract

**Background:**

Sirtuin 6 (Sirt6) is a highly conserved ADP-ribosylase and NAD+ dependent deacylase, involved in broad cellular processes. This molecule possesses contradictory roles in carcinogenesis, as it has been documented to both suppressing and augmenting tumor growth. This project aimed to explore the expression and functions of Sirt6 in diffuse large B-cell lymphoma (DLBCL), especially with regards to the regulatory role of OSS_128167, a novel small molecular inhibitor targeting Sirt6.

**Methods:**

Immunohistochemistry (IHC) was conducted to assess the expression of Sirt6 on paraffin-embedded tissues. Microarray dataset GSE32918 and GSE83632 were obtained from Gene Expression Omnibus and survival analysis was performed. Lentivirus vectors either encoding shSirt6, lvSirt6 or empty lentiviral vector were stably transfected into DLBCL cells. LY1 cell transfected with shSirt6 were performed RNA-sequencing (RNA-seq) analysis, functional enrichment analyses of gene ontology (GO) and gene set enrichment analysis (GSEA). DLBCL cells were subcutaneously injected to SCID beige mice to establish xenograft models.

**Results:**

Sirt6 is found to be overexpressed in DLBCL, and is related to poor prognosis. Sirt6-deprived DLBCL cells displayed augmented sensitivity towards chemotherapy, higher rates of apoptosis, dysfunctional cell proliferation, and arrested cell cycle progression between the G2 and M phases. Selective OSS_128167-mediated Sirt6 blockage resulted in similar anti-lymphoma effects when compared to Sirt6 knocked-down DLBCL cells. PI3K signaling along with phosphorylation of its downstream targets was reduced upon Sirt6 downregulation. Xenograft models subjected to either OSS_128167 treatment or Sirt6-knockdown showed suppressed tumor growth and lower Ki-67 level.

**Conclusions:**

These findings provide mechanistic insights into the oncogenic activity of Sirt6 in DLBCL for the first time and highlighted the potency of OSS_128167 for novel therapeutic strategies in DLBCL.

## Background

DLBCL is the most frequently encountered lymphoid malignancy in adults. It is known to be clinically and biologically heterogenous [1–4]. DLBCL is an extremely malignant disease characterized by dysregulation of cell proliferation and cells resistant to apoptosis [5]. Although 50–70% of DLBCL patients are responsive to standard rituximab based chemotherapy [6–8], the remaining relapsed or refractory patients usually die from disease progression [9–12]. Therefore, there is an urgent need to identify novel targeted treatment regimens for DLBCL patients.

Sirt6 is derived from a larger molecule group known as the mammalian Sirtuins (Sirts, Sirt1–7) family [13]. It is a highly conserved NAD + -dependent deacylase and ADP-ribosylase [14]. Through these enzymatic activities, Sirt6 is associated with a myriad of biological processes including DNA repairment, glucose/lipid metabolism, transcriptional regulation as well as telomere maintenance [15, 16]. This molecule has been associated with diabetes, heart disease, aging and cancer [17]. Evidence from recent years indicated that Sirt6 is tightly linked to the development and progression of multiple myeloma (MM) and several types of solid tumor [18, 19]. Sirt6 is a double-edged sword in carcinogenesis, as it possesses both oncogenic and tumor suppressive abilities due to the complexity of its upstream and downstream signaling pathways [20, 21]. Further investigation on the function of Sirt6 might provide new insight in the development of chemotherapeutic regimens.

Nevertheless, the impact of Sirt6 in DLBCL has yet to be fully clarified. We hypothesized that Sirt6 plays a vital role in the pathogenesis and progression of DLBCL. This study seeks to evaluate the expression pattern, functional mechanism and potential clinical relevance of Sirt6, with special focus on the regulatory role of its inhibitor OSS_128167 in DLBCL.

This series of experiments are comprised of gain- and loss-of-function assays, bioinformatics analyses, RNA-sequencing (RNA-seq) as well as xenograft models. Altogether, our study indicated that Sirt6 expression was raised in DLBCL, with its high levels corresponding to poor patient outcomes. Sirt6 was also found to promote tumorigenesis by regulating the PI3K/Akt/mTOR pathway. Targeting Sirt6 exerted anti-lymphoma activity and enhanced chemo-sensitivity. OSS_128167 may prove to be a useful component in further development of novel chemotherapy regimens in DLBCL.

## Materials and methods

### In silico analysis

The Gene Expression Omnibus was used to source microarray datasets GSE32918 and GSE83632. The R-package illumineHumanWGDASLv3.db data probe was used to annotate the GSE32918 data set before its conversion into gene symbols. The classical Bayesian method provided by the Limma package was used to carry out differential expression analysis of GSE83632 gene expression profiles before extraction of Sirt6 gene expression values was carried out.

### Clinical specimens

A total of 70 paraffin-embedded archived tissues that were previously extracted from DLBCL patients (37 females and 33 males; range of ages between 13 and 83 years, median 58 years) between 2011 to 2018 along with 35 control specimens of reactive hyperplasia lymphoid (RHL) were used for these experiments. Established clinical criterion was used to verify the diagnosis of DLBCL [22]. Healthy donors were recruited to donate peripheral blood samples, which were then subjected to the Ficoll-Hypaque density gradient centrifugation method in order to extract peripheral blood mononuclear cells (PBMCs). Primary DLBCL cells were extracted from DLBCL patients (2 females, 43 and 66 years, and 1 male, 56 years). Each human sample was obtained with informed consent in strict compliance to the Declaration of Helsinki. All study protocols were reviewed and approved by the Medical Ethical Committee of Shandong Provincial Hospital affiliated to Shandong University (SPHASU).

### Cell lines and reagents

Human primary DLBCL cells and human DLBCL cell lines LY1, LY8, Val, and LY3 were maintained in IMDM medium (Gibco, CA, USA) that contained 2 mM L-glutamine, 1% penicillin/streptomycin mixture and 10% heatinactivated fetal bovine serum (HyClone, UT, USA). These cells were left to incubate at 37 °C at a humidified 5% CO_2_ atmosphere. All human cell lines were examined for mycoplasma infection periodically and authenticated using small tandem repeat profiling conducted by LGC standards and ATCC. Nicotinamide (S1899), OSS_128167 (S8627), Doxorubicin (Adriamycin, S1208) and Bendamustine (S1212) were procured from Selleck Chemicals (TX, USA). Recombinant human IGF-1 (100–11) was purchased from PeproTech (NJ, USA).

### Cell transfection

Lentivirus vectors that encoded either shSirt6, lvSirt6 or an empty lentiviral vector (control) were constructed by GeneChem (Shanghai, China). The following RNAi sequences were used: shSirt6 1#, CGGGAACATGTTTGTGGAA; shSirt6 2#, CCGGATCAACGGCTCTATC; shSirt6 3#, CCCTGGTCTCCAGCTTAAA. DLBCL cells were infected with lentivirus vectors according to the manufacturer’s instruction at a multiplicity of infection (MOI) = 100. Stable cells were filtered at 72 h post-transfection using 5 μg/ml puromycin. The efficiencies of each transfection reaction were evaluated with a fluorescence microscope using green fluorescent protein (GFP) and further validated using western blot and qRT-PCR assays.

### RNA-sequencing

Extraction of total cell RNA was carried out with the help of RNAiso Plus (TaKaRa, Dalian, China). 1 ~ 2 μg of total RNA per sample (3 stable shSirt6 transfected and 3 shControl transfected LY1 cell sample) was utilized for constructing a KAPA Stranded RNA-Seq Library Prep Kit (Illumina) sequencing library. Afterwards, the constructed library was cross-checked using an Agilent 2100 Bioanalyzer and quantified by qRT-PCR. Lastly, the Illumina HiSeq 4000 (service provided by Kangchen Biotech, Shanghai, China) was used to further sequence the libraries that had different mixed samples.

### Immunohistochemistry (IHC) and hematoxylin-eosin (H&E) staining

IHC and H&E staining were operated according to the manufacturers’ instructions as previously illustrated [8, 23]. Detailed information was shown in Supplementary methods. Primary antibody stained human samples was anti-Sirt6 (1:160, HPA071776, Sigma, MO, USA) or anti-Ki-67 (1:200, ab15580, Abcam, Cambridge, UK).

### Quantitative real-time PCR

RNAiso Plus (TaKaRa) was used to extract total RNA. Reverse transcription reactions were carried out using reverse transcription reagents (TaKaRa). Amplification reactions were conducted using the SYBR Green Master Mix (TaKaRa) in Light Cycler 480II (Roche, Basel, Swizerland). Sirt6-specific primers used were as follows: forward, 5′-TGTGCCAAGTGTAAGACGCAG; reverse, 5′-TTGCCTTAGCCACGGTGCAG.

### Western blotting

Western blot analysis was carried out as previously interpreted [23, 24]. Primary antibodies in this experiment comprised of: anti-Sirt6 (Sigma), anti-phosphor-PI3 Kinase p110α, anti-phospho-AKT(Ser473), anti-total pan-AKT, anti phospho-mTOR, and anti-total pan-mTOR, anti-PTEN, anti-phospho-4EBP1, anti-FoxO1, anti-HIF-1α, anti-PARP [specific to the full-length (116 kDa) and the cleaved form (89 kDa) of PARP], anti-p27, anti-CDK2, anti-phospho-ATM, anti-phospho-ATR, anti-phospho-Chk1 (Ser345), anti-phospho-Chk2 (Thr68) and anti-β-tubulin (Cell Signaling Technology, MA, USA), β-actin (Zhongshan Goldenbridge, Beijing, China). Details are shown in Supplementary methods.

### Cytotoxicity assay

DLBCL cell viability was evaluated using the Cell Counting Kit-8 (CCK-8; Dojindo, Japan). DLBCL cells with designed treatment were disseminated onto 96-well plates for 24–72 h. Following this, the cells were left to incubate with 10 μl of CCK-8 per well at 37 °C for 4 h. The absorbance at 450 nm was measured using Multiskan GO Microplate Reader (Thermo Scientific, USA).

### Flow cytometry analysis

DLBCL cell apoptosis and cell cycle analysis with the designed treatments were detected by flow cytometry as previous described [23, 24]. Details are shown in Supplementary methods. All assays were carried out using the Navios Flow Cytometer (Beckman Coulter, CA, USA).

### In vivo xenograft study

Principles of Animal Care and Use Ethics Committee of SPHASU and ARRIE guidelines were strictly adhered to upon conduction of all animal experiments. 6-week-old beige female mice with severe combined immunodeficiency (SCID) were obtained from the Weitong Lihua Laboratory Animal Center (Beijing, China) and reared in a pathogen-free environment. All mice were randomly cohorted into two groups. As previously illustrated [8, 23], DLBCL cells (either transfected with shSirt6 vectors or empty control vectors) were subcutaneously injected to SCID-beige mice to establish xenograft models (*n* = 8 per group). Details are described in Supplementary methods. Experiments using OSS_128167 involved first subcutaneously injecting the SCID beige mice with 1 × 10^7^ LY1 cells. Eight days after the first injection, mice were administered with intraperitoneal injections of either OSS_128167 (80 mg/kg, *n* = 4) or a control vehicle (*n* = 4) every 2 days for 2 weeks. Pathological analysis was then carried out with dissected tumor tissues.

### Statistical analysis

All of the statistical analyses were performed using SPSS23.0 (SPSS Inc., USA) for Windows and Graphpad Prism 5.0 statistical software. Experimental data obtained from at least three separate experiments were depicted using the mean ± standard deviation (SD). Kaplan Meier analysis allowed us to plot survival curves, with the log-rank test applied for group comparison. The chi-square test with continuity correction was used to evaluate the association between clinical patient profiles and Sirt6 expression. T-tests or one-way analysis of variance (ANOVA) were used to determine the differences between experimental groups. A *p* value of less than 0.05 was interpreted as having statistical significance.

## Results

### Sirt6 was upregulated and correlated with adverse outcome in DLBCL

**Fig. 1:**
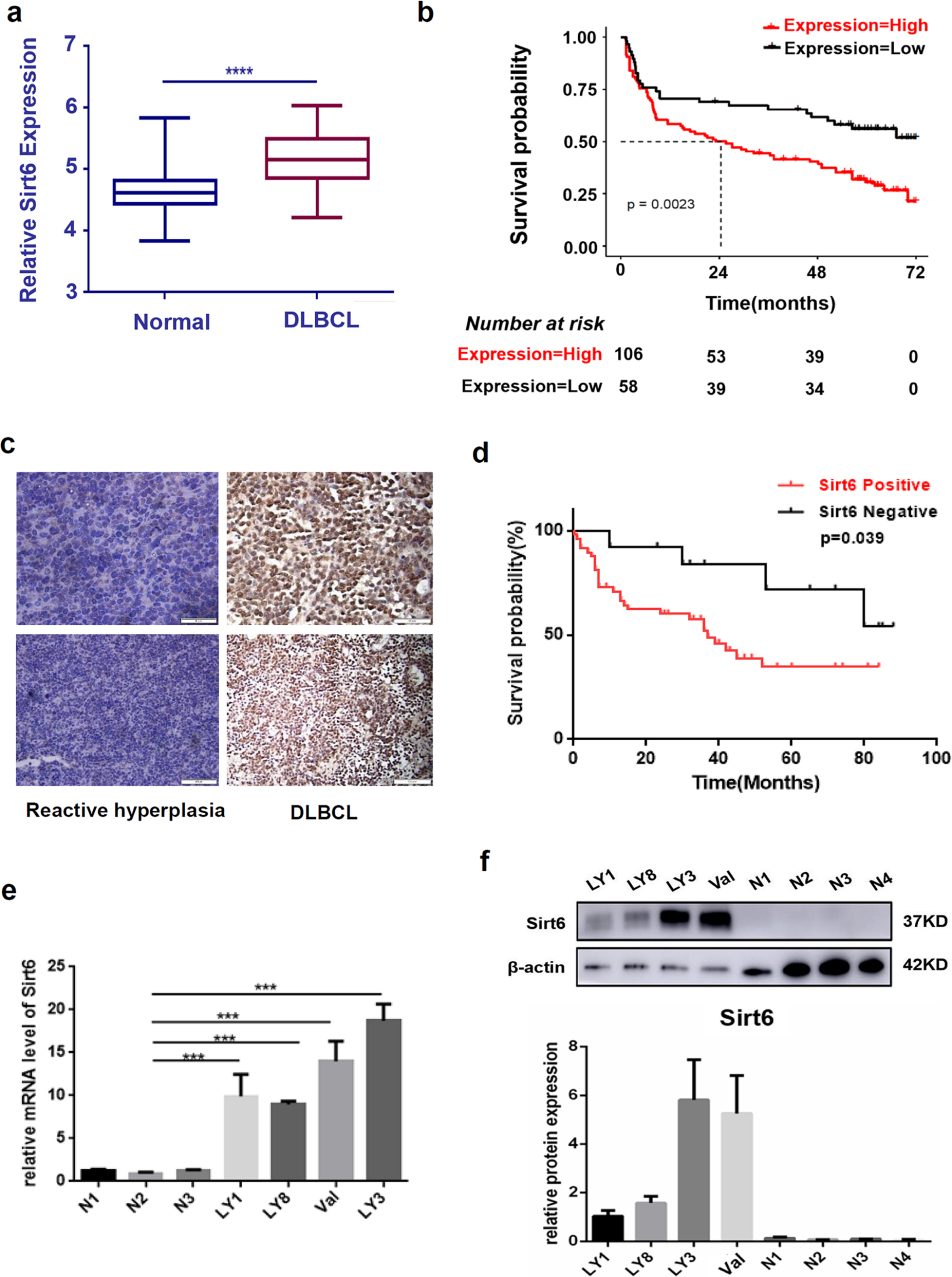
Sirt6 expressions were upregulated and correlated with adverse outcomes in DLBCL. **a** Sirt6 was upregulated in DLBCL (*n* = 163) in contrast to normal samples based on the GSE83632 data set. **b** Kaplan-Meier survival curve analysis indicated that high Sirt6 expression in DLBCL appeared to be correlated with shorter overall survival based on analysis of the GSE32918 data set (*n* = 249, *p* = 0.0023). **c** Compared to samples of RHL, expression levels of Sirt6 were significantly increased in DLBCL tissues. Upper: bar = 50 μm, lower: bar = 100 μm. **d** Kaplan–Meier survival curve analysis indicated that high Sirt6 expression in DLBCL appeared to be correlated with shorter overall survival based on analysis of our IHC data (*n* = 60, *p* = 0.039). **e** Aberrantly raised Sirt6 mRNA expression levels were observed in DLBCL cell lines (LY1, LY8, Val, LY3) compared to those in control cells (N1, N2, N3). **f** Western blotting assays showed high expression of Sirt6 protein in DLBCL cells. ***p < 0.001

To evaluate the potential role of Sirt6 in DLBCL, we first examined Sirt6 expression in GEO database. As shown in Fig. 1a, Sirt6 was markedly upregulated in DLBCL in contrast to normal samples in a bioinformatic analysis on GSE83632 (*n* = 163). Kaplan-Meier survival curve analysis of the GSE32918 indicated higher Sirt6 expression in DLBCL (*n* = 249) was correlated with shorter overall survival times (*p* = 0.0023, Fig. 1b).

There was noted to be higher Sirt6 expression levels in DLBCL tissues compared to tissues with RHL upon IHC staining (Fig. 1c), with a Sirt6 positive rate of 80% (56 of 70) in DLBCL tissues compared to 8.6% (3 of 35) in tissues with RHL. To clarify the clinical significance of Sirt6 expression in DLBCL, the clinicopathological characteristics of DLBCL patients were analyzed. Sirt6 expression levels were positively related to age (*p* = 0.009), Ann Arbor stage (*p* = 0.036), and international prognostic index (IPI, *p* = 0.045) score of DLBCL patients (Table 1). The survival analysis indicated higher Sirt6 expression in DLBCL was correlated with shorter overall survival times (*p* = 0.039, Fig. 1d). Both the mRNA and protein levels of Sirt6 were aberrantly higher in DLBCL cell lines than those in the control cells from healthy volunteers (Fig. 1e-f). These results illuminated the prognostic significance of Sirt6 in DLBCL.

**Table 1:** Correlation between Sirt6 expression and clinical characteristics of DLBCL patients

Characteristics	No. of patients	Sirt6 expression		p value
		Negative	Positive (%)	
Age(years)				
<60	41	13	28(68.3%)	**0.009**
≥ 60	29	1	28(96.6%)	
Gender				
Male	34	6	28(82.4%)	0.858
Female	36	8	28(77.8%)	
Ann Arbor stage				
I or II	35	11	24(68.6%)	**0.036**
III or IV	35	3	32(91.4%)	
B symptoms				
Present	17	2	15(88.2%)	0.531
Absent	53	12	41(77.4%)	
Sybtype				
GCB	22	6	16(72.7%)	0.479
Non-GCB	48	8	40(83.3%)	
Serum LDH				
Normal	28	9	19(67.9%)	0.077
Elevated	42	5	37(88.1%)	
Extranodal involvement				
Absent	14	2	12(85.7%)	0.823
Present	56	12	44(78.6%)	
IPI score				
0–2	41	12	29(70.7%)	**0.045**
3–5	29	2	27(93.1%)	

*LDH* Lactate dehydrogenase, *IPI* International prognostic index

### Sirt6 promoted growth of DLBCL

**Fig. 2:**
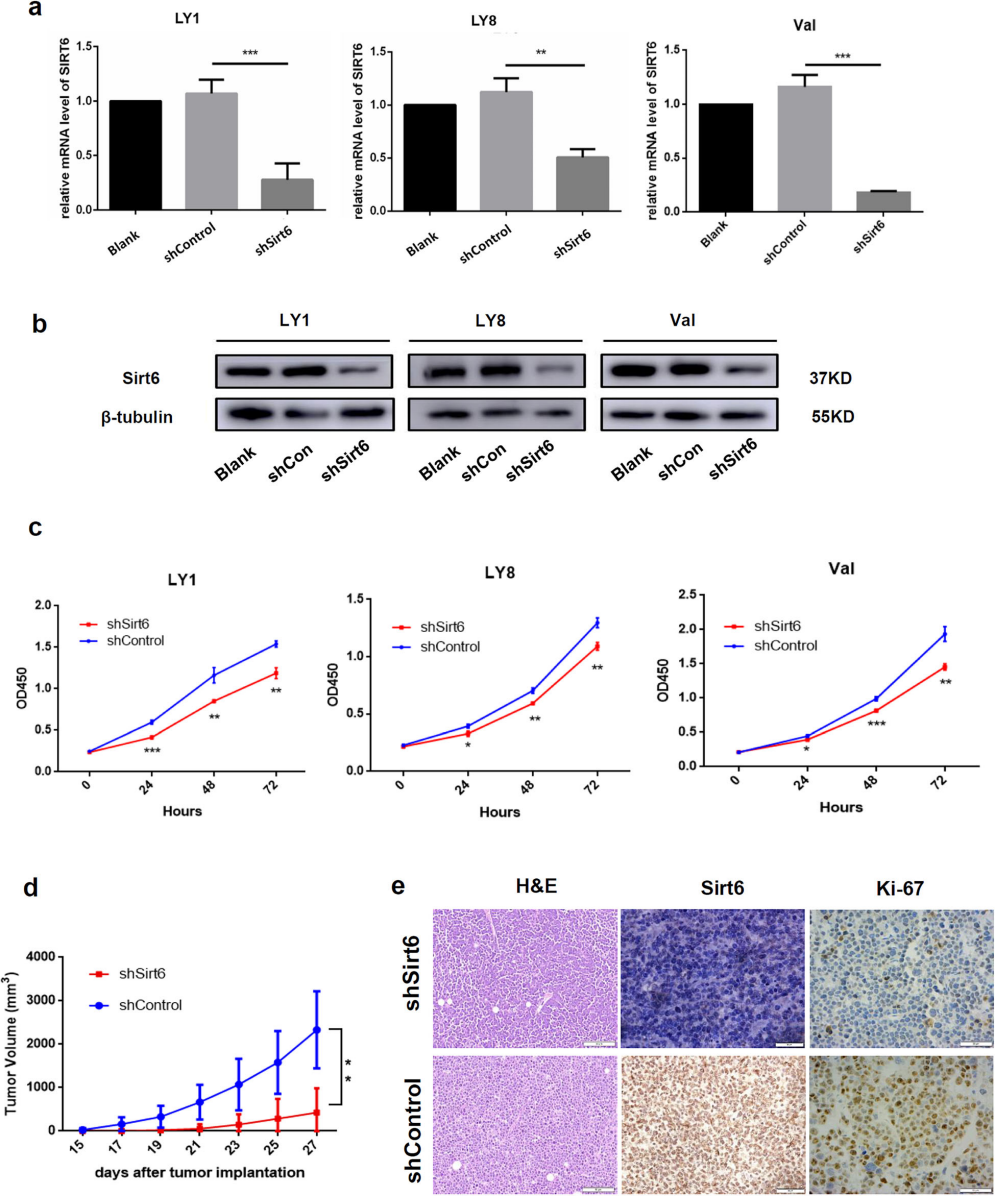
Sirt6 promoted growth of DLBCL. **a**, **b** Relative expression levels of Sirt6 were evaluated using quantitative PCR (mean ± SD, *n* = 3) and western blot in stably transfected LY1, LY8, and Val cells in contrast to empty vectors. **c** Sirt6 knockdown markedly decreased cellular proliferative activity. **d** Mice bearing shSirt6 cells were noted to have significantly reduced tumor volumes in contrast to samples transfected with empty vectors (*n* = 8 per group). **e** H&E and IHC staining of Ki-67 and Sirt6 were performed in xenograft tumor tissues. Bar = 50 μm. *p < 0.05; **p < 0.01; ***p < 0.001

The function of Sirt6 was further investigated using lentivirus mediated gain- and loss-of-function assays. Three lentivirus mediated RNA interference (RNAi) vectors carrying GFP against Sirt6 demonstrated effective knockdown of Sirt6 in human LY1, LY8, and Val cells, of which shSirt6#1 exhibited the highest efficacy. Effective knockdown (shSirt6, Fig. 2a-b) or overexpression (lvSirt6, Supplemental Fig. 1a) was verified using western blotting or qRT-PCR experiments. Stable shSirt6 transfected DLBCL cells exhibited growth suppression in contrast to cells with empty vectors (Fig. 2c). However, overexpression of Sirt6 in DLBCL cells had no impact on the proliferative ability of cells, an effect likely being the result of constitutively high Sirt6 levels (Supplemental Fig. 1b).

Furthermore, we established a mouse xenograft model using human DLBCL cells to investigate the tumor-promoting effect of Sirt6 in vivo. SCID beige mice received subcutaneous injections of either shSirt6 or shControl transfected LY1 cells (*n* = 8 per group). Mice bearing shSirt6 were found to have markedly smaller tumor volumes in comparison to the control group, which was consistent with the result obtained in our in vitro experiments (*p* < 0.01, Fig. 2d). Lower Sirt6 expression levels were confirmed in the xenograft tumor tissues derived from shSirt6 cells by IHC staining. Besides, we also observed lower expression level of proliferative marker Ki-67 [25, 26] in shSirt6 group (Fig. 2e), indicating the positive regulation of Sirt6 on DLBCL cell proliferation.

### Sirt6 inhibition promoted cell apoptosis and cell cycle arrest in DLBCL cells

**Fig. 3:**
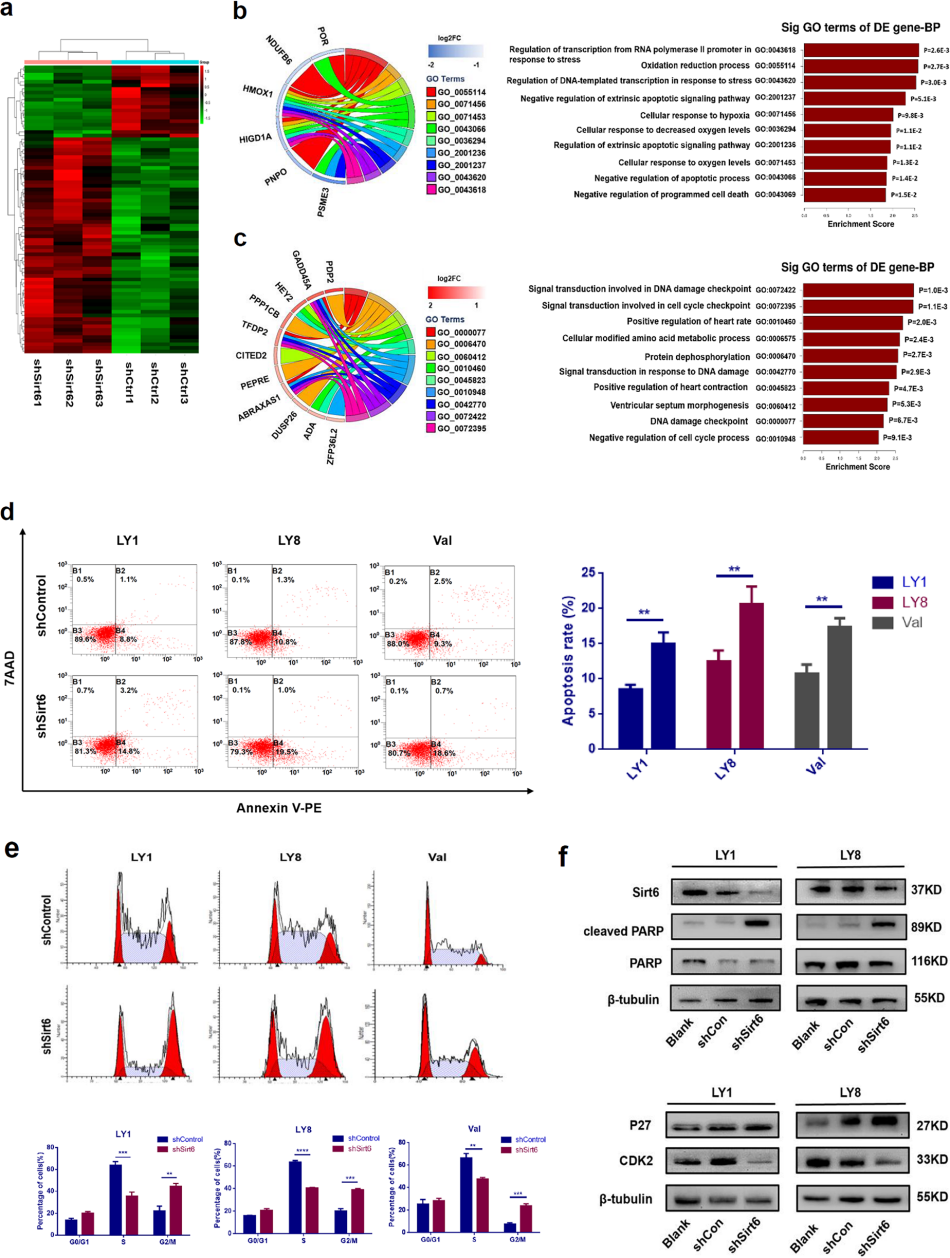
Sirt6 inhibition promoted cell apoptosis and cell cycle arrest in DLBCL cells. **a** Heatmaps of the Sirt6 correlated gene-expression signature in RNA-seq analysis. Columns represent samples and rows represent genes. **b**, **c** Functional enrichment analyses of differentially expressed genes according to RNA-seq of LY1 cells with Sirt6 knocked down, where b depicts the biological process of gene enrichment in the down-regulated group and c describes the biological process of up-regulated gene enrichment. In the GO Chord plot in **b**, the left side shows some significantly down-regulated genes (the blue color bar next to the gene represents down-regulation, and in **c** the red bar represents up-regulation), the right side shows different GO terms, and the colored ribbon connected to genes represent the genes enrichment in this GO term. The GO Chord plot on the left and the GO Enrichment score plot on the right in **b** and **c** represent the same meaning. **d** Sirt6 knockdown induced increased apoptosis rates in LY1, LY8, and Val cells as assessed by flow cytometric analysis with Annexin V-PE/7AAD staining. **e** Sirt6-depletion induced cell cycle arrest at the G2/M phase in LY1 and LY8 cells. Cell cycle distribution was detected using flow cytometry (mean ± SD, *n* = 3, **p < 0.01, ***p < 0.001). f) Decreased expression levels of CDK2 and increased levels of activated PARP and p27 were observed in sh-Sirt6-treated DLBCL cells

The pathological effects of Sirt6 in DLBCL were further explored using RNA-seq analysis on stable shSirt6 transfected or shControl transfected LY1 cells. Differentially expressed genes and transcripts were determined for further analysis. As depicted in annotations of gene ontology (GO) analysis in Fig. 3a-c, Sirt6 appeared to be strongly correlated to processes associated with cell cycle, DNA damage, and cell apoptosis.

We next verified the above Sirt6-related biological processes in DLBCL cell lines. Annexin V-PE/ 7AAD apoptotic assays indicated that shSirt6 caused higher rates of cellular apoptosis in both LY1 (8.63 ± 0.44% in Control vs.14.23 ± 0.35% in shSirt6 group, *p* = 0.0006), LY8 (10.80 ± 1.00% in Control vs. 19.03 ± 1.85% in shSirt6 group, *p* = 0.0014), and Val cells (10.73 ± 0.76% in Control vs.17.43 ± 0.69% in shSirt6 group, *p* = 0.0029, Fig. 3d). Conversely, Sirt6 overexpression resulted in reduced apoptosis (LY1: 8.63 ± 0.44% in Control vs. 4.40 ± 0.15% in lvSirt6 group, *p* = 0.0008; LY8: 10.80 ± 1.00% in Control vs. 2.77 ± 0.32% in lvSirt6 group, *p* = 0.0016, Supplemental Fig. 1c). The anti-apoptotic effect of Sirt6 was also validated by western blot analysis, as indicated by higher levels of cleaved PARP in shSirt6 cells (Fig. 3f). We then monitored cell cycle by PI staining. Knockdown of Sirt6 induced an obvious increase of DLBCL cell counts in the G2/M phase compared to cells transfected with shControl (Fig. 3e). Besides, expression of p27 and CDK2 were modestly changed in shSirt6 cells (Fig. 3f). Altogether, these results suggested that Sirt6 could promote DLBCL cell survival via acceleration of the cell cycle out of the G2/M phase and reduced rates of apoptosis.

### OSS_128167 exerted anti-tumor activity in DLBCL cells

**Fig. 4:**
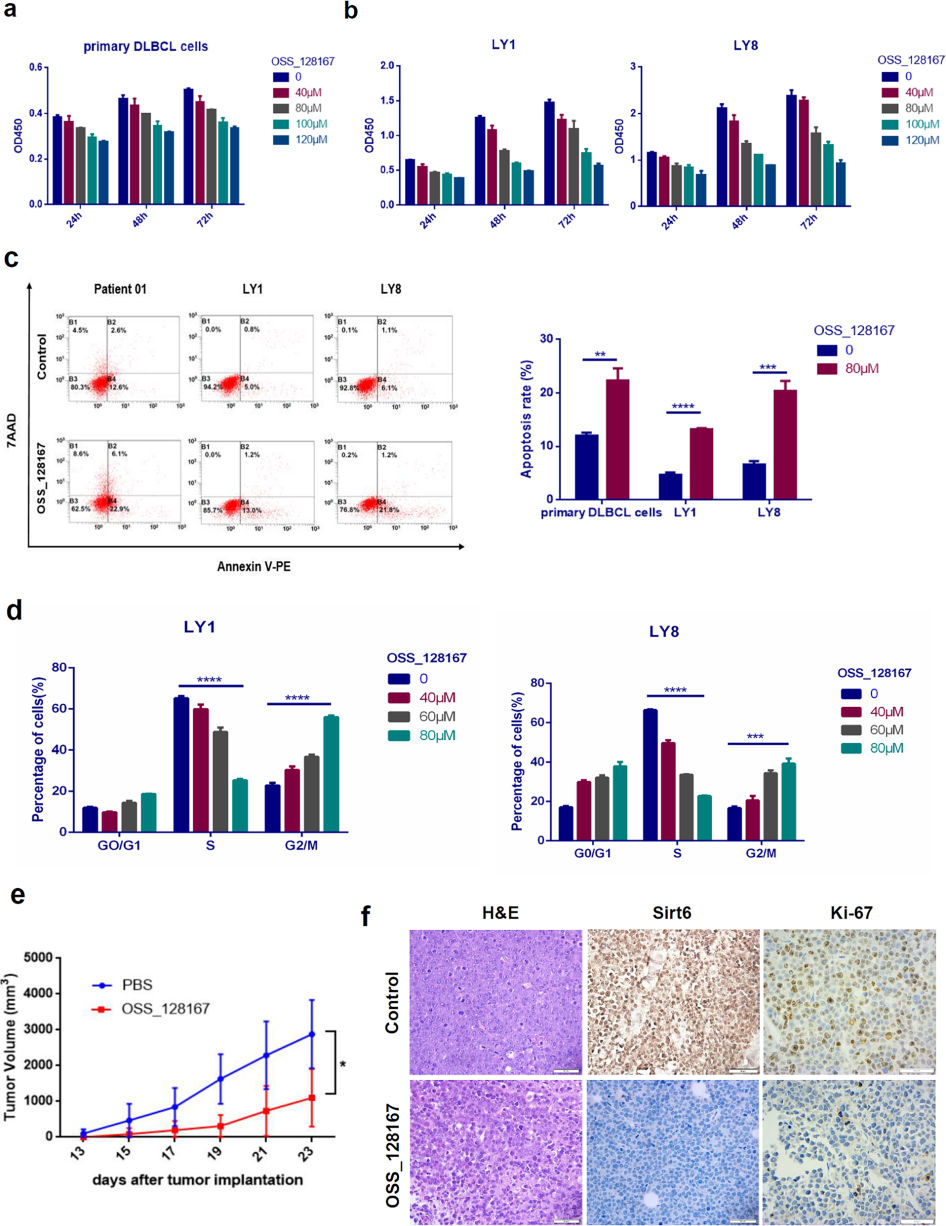
Targeted inhibition of Sirt6 by OSS_128167 exerted anti-tumor activity in DLBCL cells. **a** OSS_128167 decreased cell viability in primary DLBCL cells. **b** OSS_128167 decreased cell viability in DLBCL cell lines. **c** OSS_128167 induced fast onset cell apoptosis in primary DLBCL cells and cell lines. **d** OSS_128167 induced G2/M cell cycle arrest in DLBCL cells. **e** LY1 cells were injected subcutaneously into the SCID beige mice. Eight days after, the mice were treated with intraperitoneal injection of either OSS_128167 (80 mg/kg) or a control vehicle every 2 days for 2 weeks. Decreased tumor growth were observed in mice treated with OSS_128167 in contrast to the vehicle control group (*n* = 4 per group). **f** H&E and IHC staining with Sirt6 and Ki-67 were performed. Bar = 50 μm. *p < 0.05; **p < 0.01; ***p < 0.001; ****p < 0.0001

To further confirm the effect of Sirt6 inhibition on DLBCL, the function of OSS_128167, a novel Sirt6-specific inhibitor which can permeate cell membrane and exhibit biologically active in cultured cells, was investigated. OSS_128167 increases H3K9 acetylation and GLUT-1 expression [27]. Studies on OSS_128167 is still in the early stages given its relatively new introduction to the market. Michele Cea et al. suggested that OSS_128167 was able to sensitize primary MM cells, as well as doxorubicin- and melphalan- resistant MM cell lines, towards chemotherapy [18]. Primary DLBLC cells and DLBCL cell lines (LY1, LY8) were exposed to various concentrations of OSS_128167 for 24–72 h, respectively. OSS_128167 decreased cell viability in primary DLBCL cells (Fig. 4a). Cellular proliferation was suppressed in a time- and dose-dependent manner with a reduction by 60% noted at 48h post-exposure to a concentration of 100 μM in DLBCL cell lines (Fig. 4b).

Moreover, flow cytometric analysis revealed that the level of primary DLBCL cells and DLBCL cell lines (LY1, LY8) undergoing early apoptosis were markedly raised after incubation with 80 μM OSS_128167 for 48 h (Fig. 4b). Gradually increasing concentrations of OSS_128167 treatment also impacted the cell cycle progression of DLBCL cells. G2/M phase cell counts elevation was consistent with prior studies (Fig. 4c).

The anti-tumor impact of OSS_128167 on DLBCL cells was further investigated in vivo using a xenograft model of SCID beige mice. Consistent with our in vitro results, obviously decreased tumor growth was noted in the mice treated with OSS_128167 in contrast to the vehicle control group (Fig. 4d). Additionally, lower expression level of the proliferative marker Ki-67 was observed in OSS_128167 exposed mice (Fig. 4e). This experiment for the first time performed intraperitoneal inoculation of OSS_128167 onto mice.

### Sirt6 knockdown enhanced chemo-sensitivity in DLBCL

Doxorubicin, a DNA damaging agent, is a common agent frequently incorporated into chemotherapeutic regimens against DLBCL. Bendamustine is another DNA damaging drug used for chronic lymphocytic leukemia (CLL), MM, and more recently for non-hodgkin’s lymphoma (NHL) [28, 29]. Nevertheless, there is still a large proportion of patients that remain refractory to all these agents, representing a gap in current chemotherapeutic efficacies. Previous research has shown that Sirt6 played important roles in response to DNA damage agents and in drug resistance [18, 30–32].

**Fig. 5:**
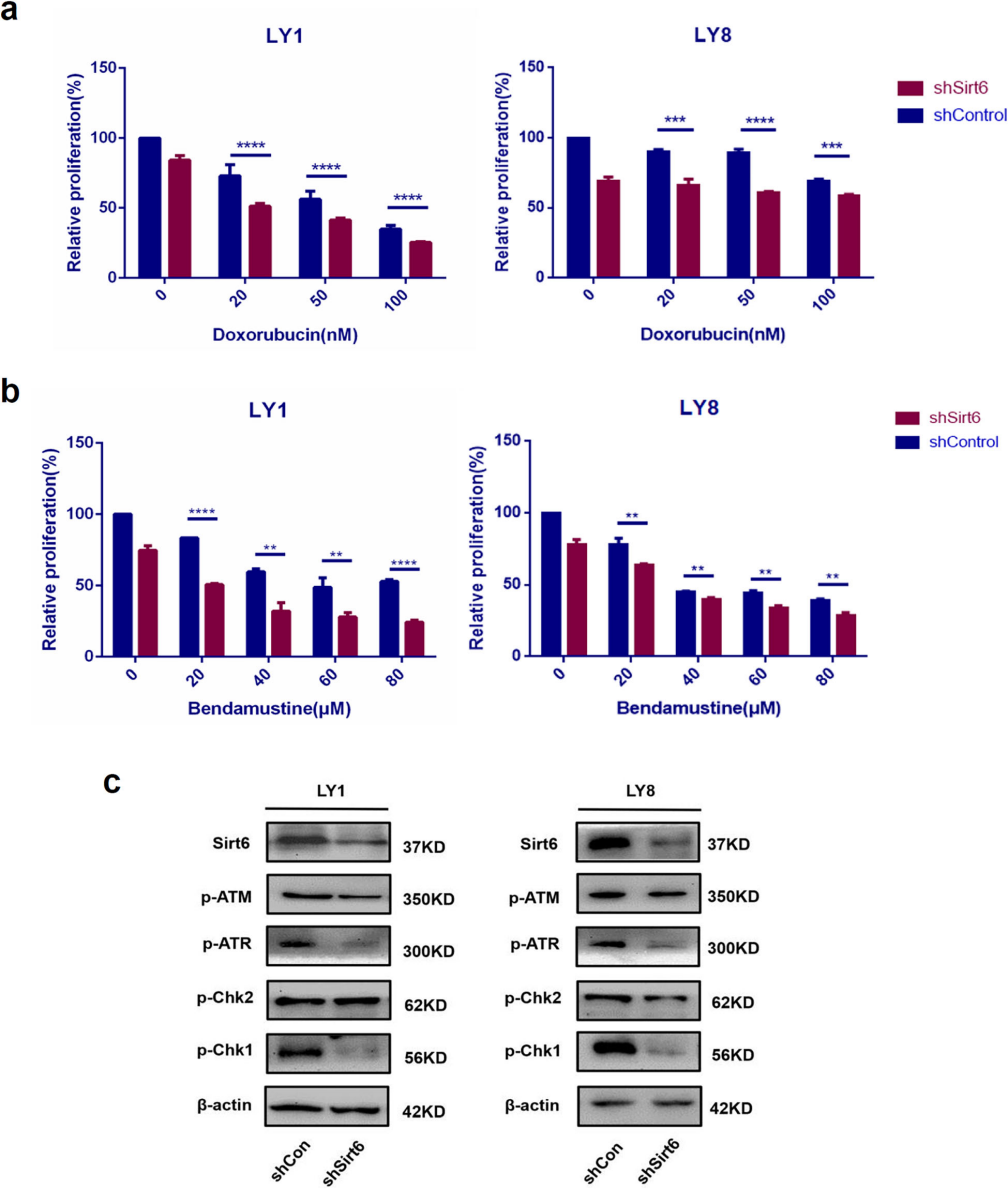
Inhibition of Sirt6 enhanced chemo-sensitivity in DLBCL samples. **a**-**b** shSirt6 cells and control cells were treated with various concentrations of doxorubicin or bendamusitine, CCK8 assay was conducted after 48 h. The cell proliferation of shSirt6 DLBCL cell lines was markedly reduced. All results are expressed as mean ± SD, *n* = 3. **p < 0.01; ***p < 0.001; ****p < 0.0001. **c** Western blot was conducted to assess the phosphorylated (p) protein levels of DNA damage signaling pathway. Cells transfected with shSirt6 were observed to have decreased expressions of phosphorylated ATR and phosphorylated Chk1

Therefore, we investigated the effects of Sirt6 in drug responses to doxorubicin and bendamustine by cell cytotoxicity assays. shSirt6 cells and control cells were exposed for 48 h to pre-determined concentrations of doxorubicin or bendamusitine. Notably, the cell proliferation of shSirt6 DLBCL cell lines was markedly reduced (*p* < 0.05, Fig. 5a-b).

DNA damage is closely related to the occurrence, progression and treatment of cancer. Cellular responses to DNA damage encompass complex mechanisms and checkpoint responses that can maintain genome stability and delay cell cycle progression. Master regulators of the DNA damage response (DDR) program are two proteins, the ataxia-telangiectasia mutated (ATM), and ataxia-telangiectasia and RAD3-related protein (ATR), which can phophorylate protein substrates [33]. The most important substrates among them are serine-threonine protein kinases- Chk1 and Chk2 [34]. In vertebrates, the ATM-Chk2 and ATR-Chk1 signaling cascades coordinated the DDR responses and cell cycle checkpoints, the latter pathway is the principal direct effector [35]. Considerable evidence has accumulated to support that, defects in DNA damage responses, especially inhibit Chk1, can result in sensitization to genotoxic stress [36–38]. Our western blotting experiments showed that, cells transfected with shSirt6 were noted to have reduced expressions of phosphorylated ATR and phosphorylated Chk1 (Fig. 5c). These findings might shine a light on the mechanism of Sirt6 knockdown or inhibition, a process that can sensitize DLBCL cells to chemotherapeutic drugs. By the way, these consequences are echoed in our RNA-seq analysis results.

### Knockdown of Sirt6 inhibited activation of PI3K/Akt/mTOR signaling pathway

**Fig. 6:**
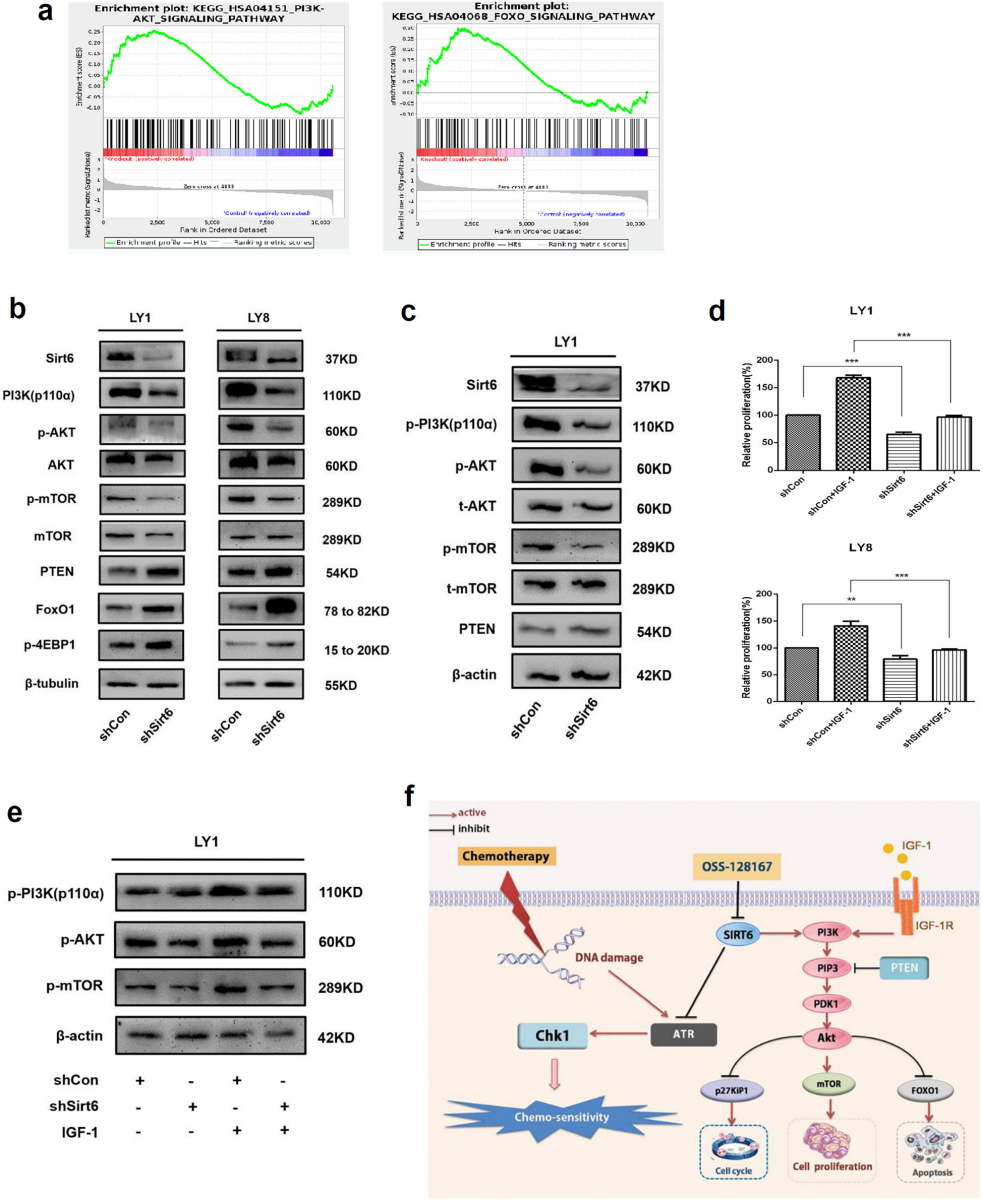
Knockdown of Sirt6 inhibited activation of PI3K/Akt/mTOR signaling pathway. **a** GSEA according to the RNA-seq implicated that Sirt6 was functionally enriched in the PI3K/Akt signaling pathway and FoxO signaling pathway. **b** Western blot was conducted to assess the phosphorylated (p) and total (t) protein levels of PI3K signaling cascades. PI3K p110α and its downstream targets, including Akt (Ser473) and mTOR protein, were observed to be dramatically decreased in Sirt6 knockdown cells in comparison to control cells. The tumor suppressor FoxO1 protein increased in shSirt6 cells. **c** Decreased activation of PI3K signaling was observed in shSirt6-treated mice. **d** IGF-1-induced DLBCL cell (LY1 and LY8) proliferation is inhibited by Sirt6-depletion. **e** LY1 cell transfected with shSirt6 or shControl, serum starved for 48 h, and treated with IGF-1. Decreased activation of PI3K signaling was observed in sh-Sirt6-treated cell. **f** Schematic description of Sirt6 mediated PI3K, FoxO, and DNA damage signaling

Next, we futher explored the potential mechanisms involved in the regulation of Sirt6, using gene set enrichment analysis (GSEA) according to RNA-seq data. Sirt6 appeared to be functionally enriched in the PI3K/Akt and FOXO signaling pathways (Fig. 6a). The conserved forkhead box class O (FoxO) transcription factor family is a prominent mediator of the PI3K signaling cascade, Akt is a key regulator of FoxO pathway [39, 40]. The FoxO proteins regulate diverse cellular and physiological processes such as apoptosis, cell proliferation, cell cycle, and DNA repair pathways [41, 42]. Therefore, FoxO1 functions as tumor suppressor in various solid tumors and B cell lymphomas, including DLBCL [43–45]. The PI3K/Akt/mammalian target of rapamycin (mTOR) pathway is a well-established pathway in carcinogenesis, including DLBCL [46, 47]. Hence, we focused on the protein levels of this signaling pathway in DLBCL cells with Sirt6 knocked down. As shown in Fig. 6b, the phosphorylation levels of PI3K p110α and its downstream targets, Akt (Ser473) and mTOR proteins, were noted to be dramatically decreased in shSirt6 cells compared to control cells. Conversely, the total Akt and mTOR protein levels were rarely significantly different between the two groups. Moreover, increased expression levels of PTEN, a negative regulator of PI3K [48], phosphorylated 4EBP1, and FoxO1 protein were found in shSirt6 cells.

As mentioned above, we established a xenograft mouse model with human DLBCL cells. LY1 cells transfected with shSirt6 or shControl cells were injected subcutaneously into the SCID beige mice. Western blotting confirmed the reduced Sirt6 expression in the xenograft tumor sections derived from shSirt6 cells. Mice bearing shSirt6 cells were noted to have lower levels of phosphorylated PI3K and its downstream targets compared to those in the shControl group (Fig. 6c).

Insulin-like growth factor-1 (IGF-1), a known activator of PI3K signaling [49], was used to further investigate the ability of Sirt6 to modify PI3K signaling in DLBCL cells. DLBCL cells transfected with either shSirt6 or shControl were exposed to IGF-1 or vehicle control in 0.5% FBS culture medium for 24–96 h. shSirt6 cells that did not receive IGF-1 treatment demonstrated lower LY8 and LY1 cellular proliferation in contrast to cells transfected with empty vectors. Cells that received IGF-1 treatment were noted to have a partial restoration in their ability to proliferate. This effect was less in shSirt6 cells in contrast to control cells (Fig. 6d).

LY1 cells were first infected with either shSirt6 or shControl, starved of serum for 48 h and subsequently exposed for 30 min to either IGF-1 50 ng/ml or vehicle control [23]. Cells that were transfected with shSirt6 were noted to have decreased levels of phosphorylated PI3K and its downstream targets, Akt and mTOR (Fig. 6e). To sum up, these findings suggest that Sirt6 may regulate the progression of DLBCL via activating the PI3K/Akt/mTOR and DNA damage response signaling pathway (Fig. 6f).

## Discussion

Sirt6 functions as a cellular pathway checkpoint controller and is responsible for the regulation of cellular proliferation and survival. Its aberrant expression has been implicated in various cancers [20, 50–52]. As alluded to above, there is controversy regarding the role of Sirt6 in tumor development. In mouse embryonic fibroblasts (MEFs), Sirt6 knockout led to tumorigenesis independent of the activation of oncogenes, suggesting that this molecule may function as a tumor suppressor [20]. Clinical sample analysis revealed that Sirt6 is down-regulated across several malignancies such as rectal and pancreatic tumors, with its level of expression strongly associated to patient prognosis [20, 50]. Sirt6 suppresses tumor growth mainly through suppression of anaerobic glycolysis and co-suppression of MYC, thereby inhibiting cancer occurrence and development. In contrast, Sirt6 has been reported to be oncogenic in breast cancer [51], prostate cancer [52], hepatocellular carcinoma, and skin cancer [53]. Knockout of Sirt6 resulted in an increased level of DNA damage and enhanced sensitivity to chemotherapy. Michele Cea et al. reported that Sirt6 was highly expressed in MM cells and closely linked to resistance against DNA damaging agents, an effect that has been attributed to MAPK/ERK2/p90RSK signaling inhibition [18].

However, there is a lack of reports on systematic examination of the expression and biological functions of Sirt6 in DLBCL, thus we aimed to document the Sirt6 expression and functions in DLBCL. Analysis of publicly available cancer microarray databases validated that Sirt6 was upregulated in DLBCL and correlated with shorter overall survival. Likewise, we found that DLBCL cell lines and tissues possess remarkably increased expressions of Sirt6 in contrast to RHL samples and PBMCs from healthy donors, and correlated with shorter overall survival as well. Sirt6 upregulation was significantly associated with older age, higher IPI score, and later Ann Arbor stage in DLBCL patients, indicating its potential of being a prognostic factor to determine which patients may possess more aggressive diseases. Taken together, these data highlighted the potential of Sirt6 as a prognostic marker in DLBCL.

Our study then demonstrated the regulatory functions of Sirt6 in DLBCL. We suggested that Sirt6 knockdown suppressed DLBCL growth both in vivo and in vitro. Xenografted DLBCL tissues grown from shSirt6 cells possessed lower rates of cells that stained positive for Ki-67 in comparison to cells that had endogenous levels of Sirt6, indicating that Sirt6 inhibition may have tumor suppressive effects.

RNA-seq analysis further indicated that regulation of Sirt6 might cause changes in the response to DNA damaging agents, cell cycle progression, and cell apoptosis. Our data showed that Sirt6 knockdown exerted therapeutic effects on DLBCL cells by modulation of the DNA damage signaling pathway, induction of G2/M cell cycle arrest, and activation of apoptosis. Sirt6 expression levels may modulate DNA damage, thereby affecting tumor resistance to DNA damage agents [18]. The pathogenesis as well as the occurrence of drug resistance in DLBCL appeared to be related to a dysfunctional apoptotic mechanism in cancer cells [54]. Sirt6 suppression in both human prostate cancer and hepatocellular carcinoma had further underscored its ability to sensitize tumors to chemotherapy and induce apoptosis [32, 52]. Management options for DLBCL that is refractory to chemotherapy or relapses of DLBCL are limited due to drug toxicity and resistance. Cells deficient in Sirt6 were found to have reduced level of phosphorylated ATR and phosphorylated Chk1, therefore were sensitized to doxorubicin and bendamustine, as demonstrated in our cytotoxicity assays. These experiments offer evidence that Sirt6 might be a potential target for the development of novel chemotherapeutic agents. Nevertheless, the evidence of Sirt6 modulation in cell cycle progression is less clear.

In addition to DNA damage, our gene set enrichment bar graph also present a high enrichment score for cellular response to hypoxia. In terms of the cellular response to hypoxia, most functional genes in the pathway are down-regulated, which means that after Sirt6 inhibition, this forced Warburg effect is reduced. Our western blot showed that after Sirt6 inhibition, Hif-1α expression was decreased (Supplemental Fig. 2), which is consistent with the results of GO analysis. Hif-1α appears to modulate multiple genes in order to activate glycolysis and repress mitochondrial respiration in a coordinated fashion. Recent studies showed that Sirt6 promoted the Warburg effect of cancer cells via upregulation of reactive oxygen species (ROS), inhibition of ROS in Sirt6-upregulated cells could rescue activation of the Warburg effect [55]. Sirt6/Hif-1α axis promotes papillary thyroid cancer progression by inducing epithelial-mesenchymal transition (EMT) [56]. We may conduct more in-depth research on it in the future.

OSS_128167 was found to significantly decrease cell proliferation, induce cell apoptosis, and inhibit cell cycle progression in DLBCL cell lines and primary DLBLC cells. In our xenograft model, OSS_128167 strongly inhibited tumor growth (Fig. 4d), a finding that was consistent with our in vitro experiments. There was also a lower expression level of the proliferative marker Ki-67 in OSS_128167 treated mice (Fig. 4e). Despite our relatively small sample size, this innovative experiment, for the first time, performed intraperitoneal inoculation of OSS_128167 into mice. In vivo investigations revealed that OSS_128167 administration or shSirt6 transfection mediated Sirt6 downregulation, both resulted in inhibition of tumor growth in xenograft models of DLBCL. In summary, our experiments pave the way for Sirt6-based drug designs for DLBCL. Further pharmacokinetic studies are still warranted to determine the ideal drug dosage and potential adverse reactions of OSS_128167 when used alone or in combination with other chemotherapeutic agents.

GSEA analyses based on the RNA-seq revealed that Sirt6 was functionally enriched in the PI3K/Akt and FoxO signaling pathway (Fig. 6a). Western blotting analysis verified that shSirt6 significantly inhibited the activation of the phosphorylation of PI3K p110α and its downstream targets. The tumor suppressor FoxO1 protein increased in shSirt6 cells (Fig. 6b). Western blotting analysis further confirmed this finding (Fig. 6c). Cells that received IGF-1 treatment were noted to have a partial restoration (Fig. 6d-e). Sirt6-induced activation of PI3K signaling may potentially be a critical cornerstone in DLBCL development. Additional studies are required to clarify the biological mechanisms and signal crosstalk involved in Sirt6 deregulation. More extensive in vivo evaluation of OSS_128167 in DLBCL mice models are ongoing and expected to be discussed in our future studies.

## Conclusion

At present, the function of Sirt6 in tumorigenesis is still controversial. Our study, for the first time, confirmed the oncogenic role of Sirt6 in the pathogenesis of DLBCL. Aberrantly high Sirt6 levels may indicate a poorer prognosis for patients with DLBCL. OSS_128167, a novel targeted inhibitor of Sirt6, exerted excellent anti-lymphoma effects via inhibiting PI3K/Akt/mTOR signaling. In addition, blockade of Sirt6 expression enhanced the sensitivity of DLBCL cells to chemotherapeutic agents. These findings provide mechanistic insights into the oncogenic activity of Sirt6 and highlight the potency of OSS_128167 for novel therapeutic strategies in DLBCL.

Summary illustrations:

Supplementary information is available at Journal of experimental & clinical cancer research’s Website.

## Supplementary information

**Additional file 1 **: **Supplemental methods.**

**Additional file 2 **: **Figure S1:** Overexpression of Sirt6 had no impact on the proliferative ability of DLBCL cells.

**Additional file 3 **: **Figure S2:** Hif-1α expression level was decreased after Sirt6 depletion.

## Data Availability

RNA-sequencing data are available at GEO under accession number GSE135914. All data generated and analyzed during this study are included in this article and its supplementary information files.

## Abbreviations

Sirt6: Sirtuin 6
DLBCL: Diffuse large B-cell lymphoma
IHC: Immunohistochemical
RNA-seq: RNA sequencing
GO: Gene ontology
GSEA: Gene set enrichment analysis
MM: Multiple myeloma
RHL: Reactive hyperplasia lymphoid
PBMC: Peripheral blood mononuclear cell
SPHASU: Shandong Provincial Hospital affiliated to Shandong University
GFP: Green fluorescent protein
MOI: Multiplicity of infection
H&E: Hematoxylin-eosin
CCK-8: Cell Counting Kit-8
SD: Standard deviation
IPI: International prognostic index
RNAi: RNA interference
CLL: Chronic lymphocytic leukemia
NHL: Non-hodgkin’s lymphoma
PI: Propidium iodide
PI3K: Phosphoinositide-3-kinase
mTOR: Mammalian target of rapamycin
FoxO1: Forkhead box protein O1
IGF-1: Insulin-like growth factor-1
MEF: Mouse embryonic fibroblasts
ROS: Reactive oxygen species
EMT: Epithelial-mesenchymal transition
