# Supplementary material for: Sirt6 promotes tumorigenesis and drug resistance of diffuse large B-cell lymphoma by mediating PI3K/Akt signaling
Source: J Exp Clin Cancer Res. 2020 Jul 25;39:142. doi: 10.1186/s13046-020-01623-w (PMC7382040; doi:10.1186/s13046-020-01623-w)
Supplement: Supplementary file 1 — Additional file 1 : Supplemental methods. [file 13046_2020_1623_MOESM1_ESM.docx]

**Additional file 1**

**Supplemental Methods**

**Clinical specimens**

A total of 70 paraffin-embedded archived tissues that were previously extracted from DLBCL patients (37 females and 33 males; range of ages between 13—83 years, median 58 years) between the years 2011 to 2018 along with 35 control specimens with reactive hyperplasia lymphoid (RHL) were used for these experiments. Established clinical criterion was used to verify the diagnosis of DLBCL.(1) Healthy donors were recruited to donate peripheral blood samples, which were then subjected to the Ficoll-Hypaque density gradient centrifugation method in order to extract peripheral blood mononuclear cells (PBMCs). Primary DLBCL cells were extracted from DLBCL patients (2 females, 43 and 66 years, and 1 male, 56 years). Each human sample was obtained with informed consent in strict compliance to the Declaration of Helsinki. All study protocols were reviewed and approved by the Medical Ethical Committee of Shandong Provincial Hospital affiliated to Shandong University (SPHASU).

**Immunohistochemistry (IHC) and hematoxylin-eosin (H&E) staining**

The paraffin in paraffin-embedded tissue samples that measured 4-μm-thick were first removed and samples rehydrated. 1x EDTA was then used to retrieve antigens under high pressure followed by a 1.5h cooling period. 3% hydrogen peroxide was then used to block endogenous peroxidase reactions before the samples were incubated with 5% BSA to nullify binding that was non-specific. Tissue sections were further left to incubate overnight with primary antibodies, anti-Sirt6 (1:160, Sigma) at 4℃. The slides were then rinsed the next morning before being allowed to incubate for another 30 minutes with secondary antibody at 37℃, proceeded with additional 30 minutes incubation with streptavidin-horseradish peroxidase complex (SABC). The slides were then stained with diaminobenzidine (DAB), counterstained with hematoxylin and mounted. The staining was scored based on the proportion of cells positive for lymphoma by two independent evaluators who were blinded to the patients’ clinical data using BX63 Olympus research microscope. A minimum of 50% of lymphoma cells that stained with Sirt6 were interpreted as positive cells. Fresh mice tumor specimens were fixed in 4% paraformaldehyde and paraffin-embedded. Sections were cut into 4-μm thickness and stained with IHC and H&E. Primary antibodies used for mice tumor staining were anti-Sirt6 (1:160, Sigma) or anti-Ki67 (1:200, Abcam).

**Quantitative real-time PCR**RNAiso Plus (TaKaRa, China) was used to extract total RNA. Reverse transcription reactions were carried out using reverse transcription reagents (TaKaRa, China). Amplification reactions were conducted using the SYBR Green Master Mix (TaKaRa, China) in Light Cycler 480II (Roche, Swizerland). SIRT6-specific primers used were as follows: forward, 5′-TGTGCCAAGTGTAAGACGCAG; reverse, 5′-TTGCCTTAGCCACGGTGCAG.

**Western blotting**

After all experimental procedures, DLBCL cells were collected, washed, and lysed in lysis buffer (Shenergy Biocolor, China) together with 1× final concentration of phosphatase inhibitor cocktail (PhosSTOP; Roche, Germany). Equal protein extracts (30μg) were then separated using TGXTM FastcastTM Acrylamide Kit polyacrylamide gels (Bio-Rad, USA) and electro blotted onto polyvinylidene fluoride (PVDF) membranes (Millipore, USA). The membranes were subjected to a 2 hour blotting period with 5% BSA at room temperature, and then subsequently an overnight blotting session at 4°C with the indicated antibodies. The following day, the PVDF membraes were TBST-rinsed and hybridized for 1 hour at room temperature with HRP-conjugated secondary antibodies (Zhongshan Goldenbridge, China). Chemiluminescent signals were evaluated with the electro-chemi-luminescence kit (Millipore, USA) with the FluorChem E imaging system (Protein Simple, USA). Primary antibodies in this experiment comprised of: Sirt6 (HPA071776, Sigma Aldrich; NB100-2522, Novus), phosphor-PI3 Kinase p110α, phospho-AKT(Ser473), total pan-AKT, phospho-mTOR, and total pan-mTOR, PTEN, phospho-4EBP1, FoxO1, HIF-1α, PARP [specific to the full-length (116 kDa) and the cleaved form (89 kDa) of PARP], p27, CDK2, phospho-ATM, phospho-ATR, phospho-Chk1(Ser345), phospho-Chk2 (Thr68) and β-tubulin (Cell Signaling Technologies), β-actin (Zhongshan Goldenbridge, China). All experiments were carried out thrice using either β-actin or β-tubulin as loading control.

**Cytotoxicity assay**

DLBCL cell viability was evaluated using the Cell Counting Kit-8 (CCK-8; Dojindo, Japan). DLBCL cells with designed treatment were disseminated onto 96-well plates for 24-72 hours. Following this, the cells were left to incubate with 10μl of CCK-8 per well at 37℃ for 4 hours. The absorbance at 450 nm was measured using Multiskan GO Microplate Reader (Thermo Scientific, USA).

**Flow cytometry analysis**

DLBCL cell apoptosis with the designed treatments were detected using a Annexin V-PE/7-aminoactinomycin (7AAD) apoptosis detection assay (BD Biosciences, USA) in compliance to manufacturer’s protocols. After washing twice using ice cold PBS, the collected cells were left to incubate in 1× binding buffer and subsequently stained in the dark for 15 minutes using 5μl Annexin V-PE and 5μl 7AAD. The percentages of cells that underwent apoptosis were determined by flow cytometry. To perform cell cycle analyses, DLBCL cells were collected and rinsed with PBS before being fixated overnight at -20°C with 70% ethanol. The cells were stained the next day in the dark for 30 minutes with PI/RNase Staining Buffer (BD Biosciences, USA). All assays were carried out using the FACS- 240 Navios Flow Cytometer (Beckman Coulter Inc. USA).

***In vivo* xenograft study**

Principles of Animal Care and Use Ethics Committee of SPHASU and ARRIE guidelines were strictly adhered to upon conduction of all animal experiments. 6-week-old beige female mice with severe combined immunodeficiency (SCID) were obtained from the Weitong Lihua Laboratory Animal Center, China and reared in a pathogen-free environment. All mice were randomly cohorted into two groups. 5 × 10^6^ LY1 cells (either transfected with shSirt6 vectors or empty control vectors) that were reconstituted with 100μl Matrigel (BD Biosciences, USA), were injected subcutaneously into the mice. Tumor volumes were recorded manually used a digital caliper every 2 days. Experiments using OSS_128167 involved first subcutaneously injecting the SCID Beige mice with 1 × 10^7^ LY1 cells. 8 days after the first injection, mice were administered with intraperitoneal injections of either OSS_128167 (80mg/kg, n=4) or a control vehicle (n=4) every two days for 2 weeks. The volume of tumor was approximated with the following formula: V = (a × b^2^) × 0.5236, where ‘a’ is the largest dimension and ‘b’ is the perpendicular diameter. The investigator was blinded to the group allocation when assessing the outcome. Pathological analysis was then carried out with dissected tumor tissues.

**References**

1. Quintanilla-Martinez L. The 2016 updated WHO classification of lymphoid neoplasias. Hematol Oncol. 2017;35 Suppl 1:37-45.
