# Supplementary material for: Sirt6 promotes tumorigenesis and drug resistance of diffuse large B-cell lymphoma by mediating PI3K/Akt signaling
Source: J Exp Clin Cancer Res. 2020 Jul 25;39:142. doi: 10.1186/s13046-020-01623-w (PMC7382040; doi:10.1186/s13046-020-01623-w)
Supplement: Supplementary file 2 — Additional file 2 : Figure S1: Overexpression of Sirt6 had no impact on the proliferative ability of DLBCL cells. [file 13046_2020_1623_MOESM2_ESM.docx]

**Additional file 2**

**Figure S1**


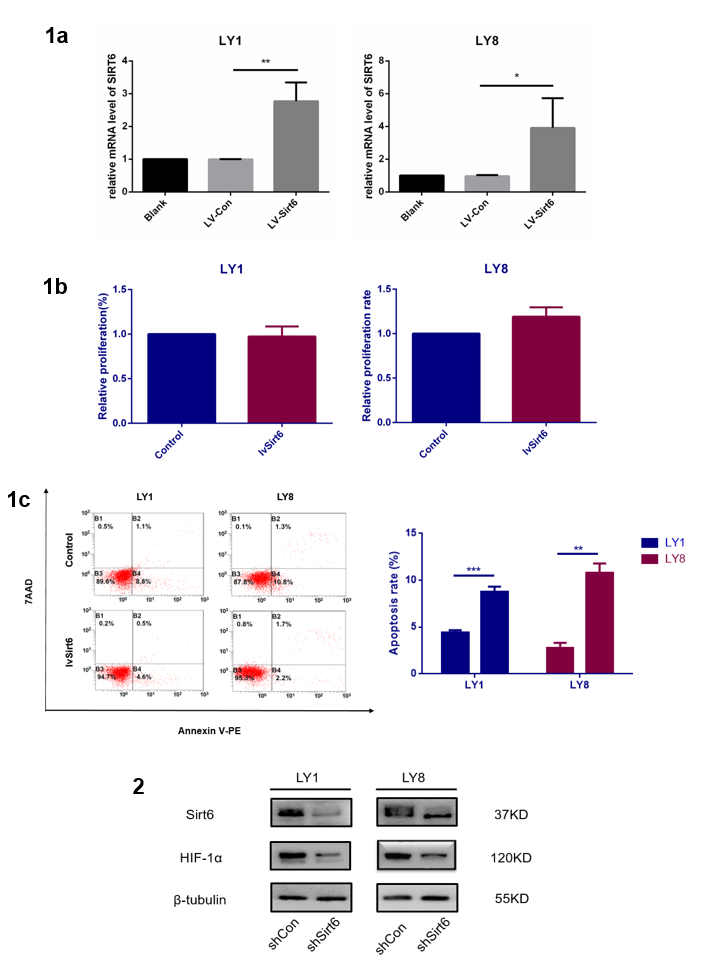


**Figure legend:**

Figure S1. Overexpression of Sirt6 had no impact on the proliferative ability of DLBCL cells. a) Effective overexpression of Sirt6 (lvSirt6) was verified in DLBCL cells using qRT-PCR experiments. b) Overexpression of Sirt6 in DLBCL cells had no impact on the proliferative ability of cells. p>0.05. Experimental data obtained from three separate experiments, depicted using the mean ± standard deviation (SD). c) Sirt6 overexpression resulted in reduced apoptosis (LY1: 8.63 ± 0.44% in Control vs. 4.40 ± 0.15% in lvSirt6 group, p= 0.0008; LY8: 10.80 ± 1.00% in Control vs. 2.77 ± 0.32% in lvSirt6 group, p= 0.0016.
