# Supplementary material for: Sirt6 promotes tumorigenesis and drug resistance of diffuse large B-cell lymphoma by mediating PI3K/Akt signaling
Source: J Exp Clin Cancer Res. 2020 Jul 25;39:142. doi: 10.1186/s13046-020-01623-w (PMC7382040; doi:10.1186/s13046-020-01623-w)
Supplement: Supplementary file 3 — Additional file 3 : Figure S2: Hif-1α expression level was decreased after Sirt6 depletion. [file 13046_2020_1623_MOESM3_ESM.docx]

**Additional file 1**

**Figure S2**


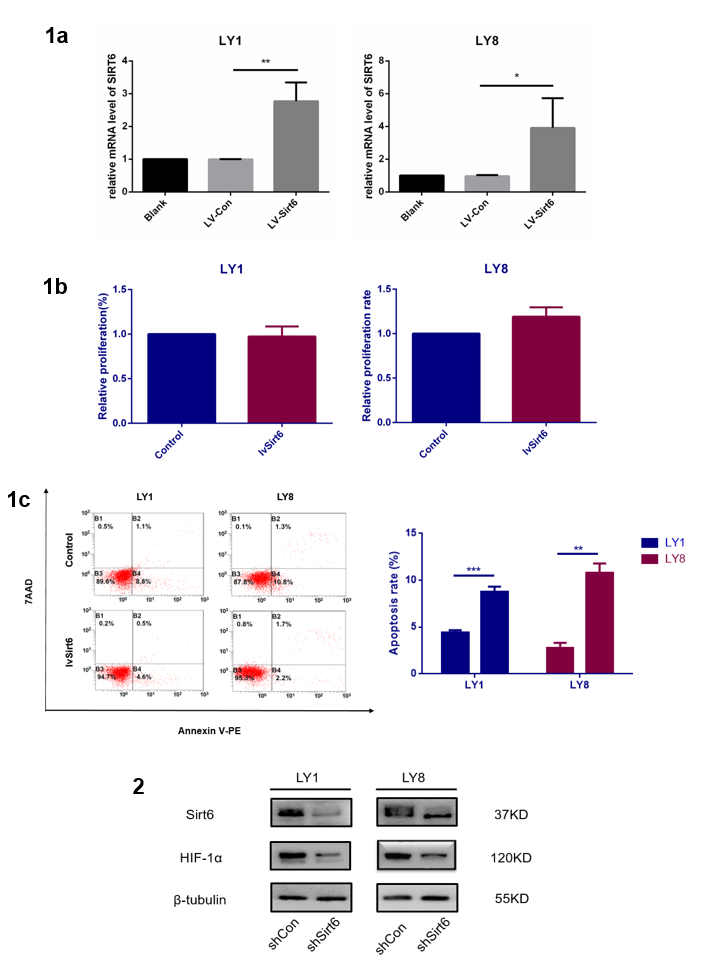


**Figure Legend:**

Figure S2. Hif-1α expression level was decreased after Sirt6 depletion.
